# Targeting Tau to Treat Clinical Features of Huntington's Disease

**DOI:** 10.3389/fneur.2020.580732

**Published:** 2020-11-19

**Authors:** Maria Masnata, Shireen Salem, Aurelie de Rus Jacquet, Mehwish Anwer, Francesca Cicchetti

**Affiliations:** ^1^Centre de Recherche du CHU de Québec, Axe Neurosciences, Québec, QC, Canada; ^2^Département de Psychiatrie & Neurosciences, Université Laval, Québec, QC, Canada; ^3^Département de Médecine Moléculaire, Université Laval, Québec, QC, Canada

**Keywords:** tauopathy, tau hyperphosphorylation, cognitive deficits, tau-targeting treatments, tau aggregation inhibitors, tau immunotherapy, microtubule stabilizers, gene silencing

## Abstract

Huntington's disease (HD) is an autosomal dominant neurodegenerative disorder characterized by severe motor, cognitive and psychiatric impairments. While motor deficits often confirm diagnosis, cognitive dysfunctions usually manifest early in the disease process and are consistently ranked among the leading factors that impact the patients' quality of life. The genetic component of HD, a mutation in the huntingtin (*HTT)* gene, is traditionally presented as the main contributor to disease pathology. However, accumulating evidence suggests the implication of the microtubule-associated tau protein to the pathogenesis and therefore, proposes an alternative conceptual framework where tau and mutant huntingtin (mHTT) act conjointly to drive neurodegeneration and cognitive dysfunction. This perspective on disease etiology offers new avenues to design therapeutic interventions and could leverage decades of research on Alzheimer's disease (AD) and other tauopathies to rapidly advance drug discovery. In this mini review, we examine the breadth of tau-targeting treatments currently tested in the preclinical and clinical settings for AD and other tauopathies, and discuss the potential application of these strategies to HD.

## Huntington's Disease: a Secondary Tauopathy

Tau is an important microtubule-associated protein primarily expressed in neurons and known to mediate a plethora of cellular functions such as microtubule dynamics, neurite outgrowth, intracellular trafficking and synaptic plasticity (1–3). These activities are tightly regulated by post-translational modifications of tau, including phosphorylation and dephosphorylation (4–6), acetylation (7, 8), glycosylation (9), O-GlcNAcylation (10), nitration (11), sumoylation (12) and truncation (13). However, while phosphorylation is an essential mechanism regulating the biological activities of tau, abnormally hyperphosphorylated tau (p-tau) can form neurofibrillary tangles (NFTs) and/or neuropil threads (NTs) (14) that interfere with these fundamental mechanisms. This has been classically associated with Alzheimer's disease (AD) or with diseases caused by mutations of the tau gene (*MAPT*) such as Frontotemporal dementia with parkinsonism-17 (15, 16) (Table 1). More recently, similar tau dysregulations have been reported in Huntington's disease (HD) (27, 31, 32, 42, 55, 64, 83, 84) [reviewed in (33, 85)]; a disorder driven by an autosomal dominant pattern of inheritance and caused by a pathological CAG repeat expansion exceeding 35 in exon 1 of the huntingtin (*HTT*) gene (86) coding for the huntingtin (HTT) protein. This CAG elongation leads to the production of mutant huntingtin (mHTT) (87–90) which confers a cytotoxic activity to this newly formed protein. This includes sequestration of transcription factors, mitochondrial dysfunction, induction of apoptotic cell death and alteration of the ubiquitin-proteasome system (UPS) (88–90).

**Table 1:** Tau pathology and therapeutic strategies in AD and HD.

***Evidence of tau dysfunction***		***Target***	***Therapeutic approach***
***Alzheimer's disease***	***Huntington's disease***		
• A90V (17), G213R (18), K280del (19), A152T (20), V287I (18), A297V (18), S318L (18), A41T (21). • H1 and H1c haplotypes may be risk factors for AD (22–25); H2 haplotype may be protective against AD (23).	• No *MAPT* mutations identified in GWAS studies (26). • Accelerated cognitive decline in H2 haplotype carriers (27).	*MAPT* polymorphism and mutations	**Modulation of** ***MAPT*** **gene expression**
• Tau tangles composed of 3R and 4R tau (28). • Shift from 4R to 3R tau-enriched NFTs in hippocampus (29). • ↑ 3R tau in brainstem with disease progression (30).	• Cortex and striatum of HD patients: ↑ 4R/3R mRNA ratio (31, 32); ↑ 4R/3R tau protein (27, 31–33); ↓ 3R tau protein (27, 31). • Cortex and striatum of R6/1 and HD94 mice: ↑ 4R/3R mRNA ratio; ↑ 4R and ↓ 3R tau protein (31).	Tau isoforms	
• Tau aggregates associated with severity of symptoms and disease progression (34, 35). • Presence of NFTs in several AD brain regions: transentorhinal region, hippocampus, neocortex (36). • Transcellular propagation of tau *in vitro* (37, 38) and spread between brain regions *in vivo* (39–41). • Propagation and deposition of tau inclusions in a sequential pattern in AD patients, from transentorhinal (stage I) to the isocortex (stage V–VI) (36).	• Presence of NFTs in post-mortem HD brain tissue (27, 42–46). • Acquisition of tau inclusions, NFTs, NTs and increased 4R/3R tau in healthy fetal neural allografts in HD recipients (42).	Tau pathological deposits and propagation	**Inhibition of tau aggregation and/or tau immunotherapies**
• GSK-3β (47–49), CDK5 (49, 50), PKA, Erk1/2, JNK1/2/3, p38, CK1 (51), TTBK1, DYRK1A (52, 53) MARK, PKB, PKC, CaMKII, SFK, c-Abl [all reviewed in (54)].	• GSK-3β (55, 56), CDK5 and CaMKII (56).	Tau-targeting kinases	**Targeting hyper-p-tau**
• PP1, PP2A, PP2B, PTEN, PP5 (57–60).	• PP1 and PP2A (56), PP2B (56, 60)	Tau-targeting phosphatases	
• Tau phosphorylation in AD brain tissue: S202, T231, S199, Y18, S262, S356 [For a detailed account on phosphorylation sites, see (61, 62)]. • ↑ phosphorylation at Y18, T231 and S199 in post-mortem brain tissue (63). • ↑ phosphorylation at Y18 and T231 in isocortex and transentorhinal cortex depending on Braak stages (63).	• Tau phosphorylation in HD brain tissue: S396, S404, T205, S199 (32) and S202 (AT8 antibody) (42, 43). • ↑ p-tau in cortex and striatum of HD patients (27, 32). • Detection of hyper-p-tau S202 and T205 in healthy fetal neural allografts in two HD patient recipients (42). • ↑ hyper-p-tau in the brains of R6/2 (56, 64), zQ175 (64) and 140CAG knock-in (56) mouse models of HD.	Disease-associated p-tau sites	**Targeting hyper-p-tau and/or tau immunotherapies**
• Prevention of *in vitro* microtubule assembly (65) and depolymerization (66, 67) by p-tau isolated from human AD brain tissue. • Reduced microtubule density and axonal degeneration in neuronal cultures (68, 69), in Tg mice expressing h-tau (70) or PS19 tau (71), and in AD patients (72). • Contribution of tau-dependent loss of microtubule stability to cognitive deficits in tau 3xTg and rTg4510 mouse models (73). • Tau-related microtubule destabilization is accompanied by Aβ-induced neurodegeneration (74–77).	• Binding of mHTT to microtubules, defects in axonal transport, mitochondrial and vesicular dynamics in primary neurons (78–81). • Reduction of BDNF axonal transport by mHTT in NG108-15 cells, resulting in neuronal loss (82). • Recruitment of mHTT to microtubules by tau (56)	Microtubule dysfunction	**Stabilizing microtubules**

*Aβ, Amyloid-beta; AD, Alzheimer's disease; BDNF; brain-derived neurotrophic factor; CaMKII, Ca^2+^/calmodulin-dependent protein kinase II; CDK5, cyclin-dependent kinase-5; CK1, Casein kinase 1; DYRK1A, Dual specificity tyrosine-phosphorylation-regulated kinase 1A; Erk1/2, extracellular signal-regulated protein kinases 1 and 2; GSK-3ß, glycogen synthase kinase-3; h-tau, human tau; HD, Huntington's disease; hyper-p-tau, hyperphosphorylated tau; JNK1/2/3, c-Jun N-terminal Protein Kinase 1/2/3; MARK, MAP/microtubule affinity-regulating kinase; mHTT, mutant huntingtin; mRNA, messenger RNA; NFTs, neurofibrillary tangles; NTs, neuropil threads; p-tau, phosphorylated tau; PKA, protein kinase A; PKB, protein kinase B; PKC, protein kinase C; PP1, protein phosphatase 1; PP2A, protein phosphatase 2A; PP2B, protein phosphatase 2B; PP5, serine/threonine-protein phosphatase 5; PTEN, phosphatidylinositol-3,4,5-trisphosphate 3-phosphatase; S, serine; SFK, Src Family Kinase; T, threonine; Tg, transgenic; TTBK1, tau-tubulin kinase 1; Y, tyrosine*.

While the diagnosis of HD is based on motor features (typically chorea), patients exhibit early and progressive cognitive impairments that impact activities of daily living along with psychiatric disturbances that can evolve to frank psychosis (91, 92). Notably, carriers of the H2 *MAPT* haplotype allegedly experience more rapid cognitive decline than those with an H1 haplotype (27). This is of particular interest since the *MAPT* haplotype has been proposed as a risk factor for other neurodegenerative disorders such as AD, Parkinson's disease (PD) and PD-associated dementia (22, 23, 93). Along these lines, data collected by Positron Emission Tomography (PET) have allowed to establish correlations between tau and cognitive decline, with tau deposits more closely related with cognitive dysfunction in AD patients than amyloid β (Aβ) (94). Furthermore, both PET and cerebrospinal fluid (CSF) measures of tau, but not Aβ, have been linked to worsening cognition in AD (95). Similarly, the CSF of HD patients contains increasing levels of total tau (t-tau) with disease progression, which correlate with a decline in motor and cognitive functions (96). While a couple of studies have found discrepancies between the levels of CSF t-tau and cognitive decline (96, 97), a correlation between CSF t-tau and mHTT has been reported (97).

In agreement with the concept that HD meets the criteria of a secondary tauopathy is the fact that the cardinal features of tauopathies—misfolding, hyperphosphorylation, NFTs and NTs—have all been identified in post-mortem brain tissue derived from HD patients (27, 43–46, 98–100) [reviewed in (33, 85)]. For example, an increased 4R/3R tau isoform ratio has been observed in *HTT* mutation carriers (31, 32) at late disease stages (3 and 4) (32). In particular, nuclear rod-like tau deposits composed of the 4R tau isoform are more abundant in striatal and cortical tissues of HD patients, while they are virtually undetectable in the brains of control individuals (31). Both abnormal p-tau and mHTT aggregates can be located within neurons (27), although they rarely colocalize (98) or co-precipitate in HD brain homogenates (31).

**Figure 1:**
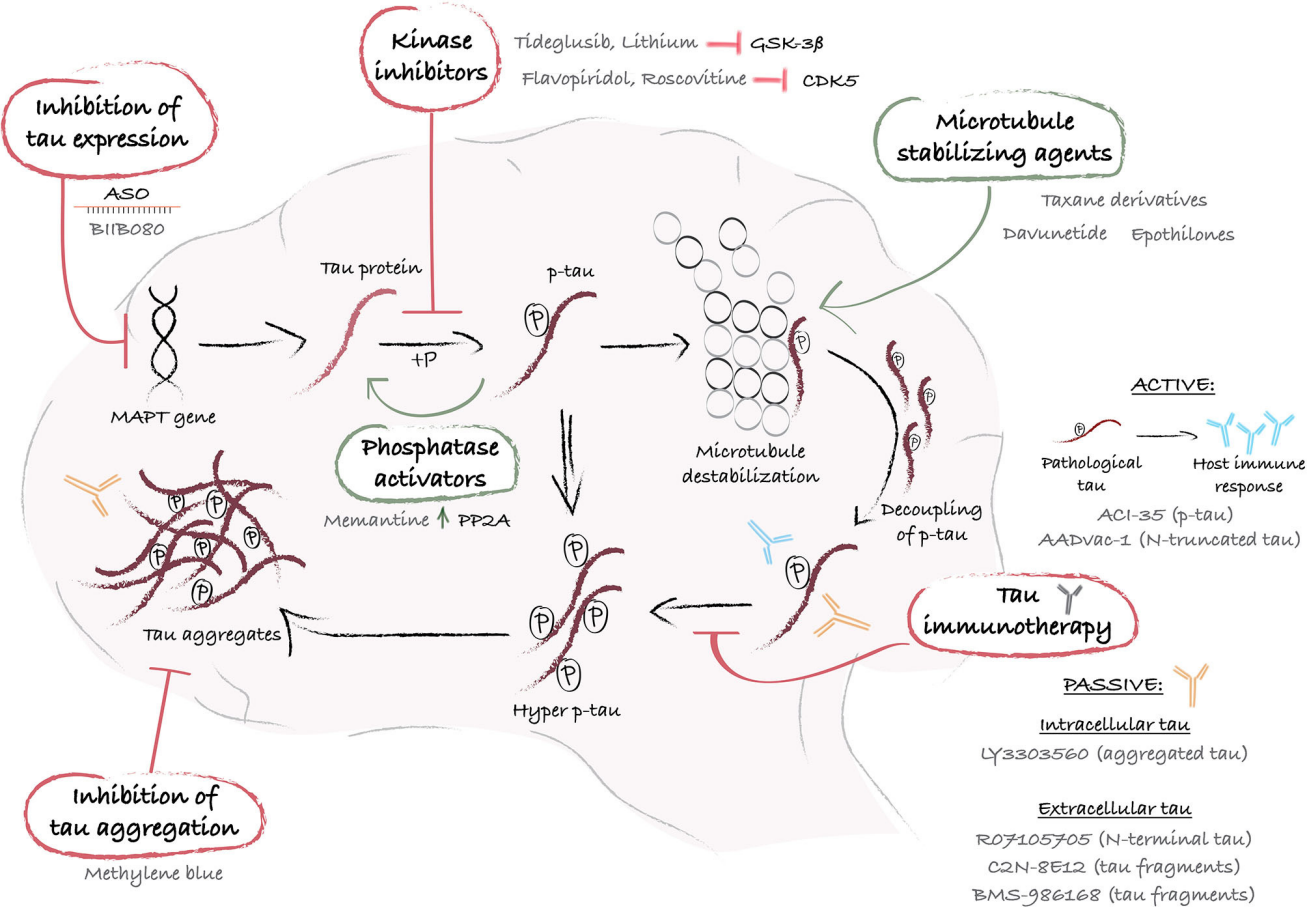
Schematic representation of mechanistic interventions using tau-targeting therapies. The *MAPT* gene encodes for the protein tau, which undergoes post-translational phosphorylation and dephosphorylation that regulate its affinity for microtubules and ensure its functional role as a microtubule stabilizer. When tau undergoes hyperphosphorylation, generally via an abnormal stimulus, it loses its affinity for microtubules and accumulates into the cytoplasm or exits the cells. When released from the microtubules, intracellular hyperphosphorylated tau proteins self-assemble into aggregates of increasing complexity, and ultimately produce large pathological aggregates and NFTs. At each step of the process, drug targets have been identified and a variety of therapeutic approaches have been designed and tested in preclinical and/or clinical studies. A first strategy leverages the use of ASO (e.g. BIIB080) to control *MAPT* gene expression and reduce tau synthesis. Alternatively, pathological tau hyperphosphorylation can be reversed with kinase inhibitors (e.g. Tideglusib or lithium) or phosphatases activators (e.g. Memantine), and tau loss of function can be counterbalanced with microtubule stabilizers (e.g. Taxane derivatives, Davunetide, or Epothilones). Furthermore, immunization-based strategies offer an interesting approach to sequester and degrade p-tau before aggregation, using peptides that mimic a specific p-tau amino acid sequence (e.g. ACl-35 or AADvac-1), active or passive immunization to target either intracellular (e.g. LY3303560) or extracellular tau (e.g. RO7105705, C2N-8E12 or BMS-986168). Lastly, tau aggregation inhibitors, such as Methylene blue, provide an additional means by which to eliminate tau aggregates and restore cellular health. ASO, antisense oligonucleotide; CDK5, cyclin-dependent kinase-5; GSK-3ß, glycogen synthase kinase-3; hyper p-tau, hyperphosphorylated tau; MAPT, microtubule-associated protein tau; P, phosphate; PP2A, protein phosphatase 2A; p-tau, phosphorylated tau.

Collectively, these findings suggest an association between altered tau biology and HD pathology. However, whether tau impairments have a causative effect on the manifestation of certain aspects of the disease, such as cognitive decline, has yet to be established. A closer look at the evidence of tau dysfunction in HD allows us to explore rather uncharted territories in therapeutic development for this condition. Taking advantage of the discoveries and therapeutics designed to attenuate tau dysfunction in AD (101), as a significant number of preclinical studies and clinical trials have already been initiated, may indeed prove to be useful in HD as well. There is a broad diversity of approaches (Figure 1), which include decreasing tau phosphorylation, inhibiting tau aggregation and reducing pathological forms of tau using microtubule stabilizing compounds, immunotherapies or silencing of the *MAPT* gene (Figure 1), which could all serve treatment purposes. In the following sections, we present the multiple therapeutic approaches to target tau, describe the treatments that have reached clinical trials and discuss their potential application to HD.

## Therapeutic Strategies to Target Pathological Forms of Tau

### Targeting Tau Hyperphosphorylation

Tau function depends on its phosphorylation state. Hyperphosphorylation generates negatively charged repulsive forces which impede functional interactions with microtubules, leading to their destabilization and subsequent cell death (5, 6, 102). Recent studies have reported an increase in p-tau levels in the cortex and striatum of post-mortem brain tissue derived from HD patients (27, 32). More specifically, hyperphosphorylation of tau has been detected at 5 distinct epitopes—S396, S404, T205, S202, and S199 residues—(32, 42, 43), and S202 and T205 were found in neuronal inclusions (27) (Table 1). The association of tau hyperphosphorylation in various forms of tauopathies (4, 103, 104), as well as in HD, suggests that approaches aiming at restoring normal levels of p-tau could improve disease outcome (Figure 1); approaches that have already been extensively tested in AD models (105–108). One of the strategies to reduce p-tau levels is to modulate the activity of tau-targeting kinases and phosphatases. The physiological function of the tau protein is facilitated by the orchestrated activity of these enzymes through phosphorylation and dephosphorylation at the threonine and serine residues (109). A number of small molecule inhibitors of protein kinases or activators of phosphatases have been studied in pre-clinical and clinical settings and are presented in the following section.

#### Kinase Inhibitors

Small molecules, which have the ability to reduce tau hyperphosphorylation, have been among the first tau-targeted treatments developed for AD. This evolved from the evidence that kinases, such as cyclin-dependent-like kinase 5 (CDK5) and glycogen synthase kinase 3 beta (GSK-3β), are altered in patients and animal models of the disease (49, 110–114) (Table 1). In a transgenic (Tg) HD mouse model that expresses exon 1 of the human *HTT* gene, with approximately 125 CAG repeats (R6/2) (115), a knock-in chimeric HD mouse model, that expresses a human *HTT* exon 1/ mouse *Htt* with 140 CAG repeats (KI140) (116), as well as in patients, the levels and activity of CDK5 and GSK-3β have been found to be dysregulated (55, 56), suggesting that redirecting kinase inhibitors levels/activity could potentially abrogate tau hyperphosphorylation observed in HD.

CDK5 inhibitors, such as Flavopiridol (Alvocidib) and Roscovitine (Seliciclib), have been tested in AD preclinical studies. Flavopiridol efficiently inhibits CDK5 and improves synaptic plasticity as well as motor behavior in mice injected with Aβ oligomers (117). Roscovitine (Seliciclib) prevents tau phosphorylation in a Niemann-Pick Type C disease mouse model (118), which is characterized by CDK5 dysfunction that triggers tau hyperphosphorylation (119). However, CDK5 inhibitors have yet to be tested in the clinical setting and further investigation is therefore needed to determine if they can, in effect, ameliorate tau-associated pathology in either, or both, AD and HD.

GSK-3β inhibitors form another class of compounds, which have been tested in preclinical and clinical contexts. Among the GSK-3β inhibitors, Tideglusib and lithium have shown improvements in AD Tg mouse models that express either four familial AD mutations (FTDP-17 G272V, P301L, and R406W, referred to as VLW mice and VLW mice overexpressing GSK-3β) (120, 121), three familial AD mutations (APP Swedish, MAPT P301L and PSEN1 M146V) (3xTg-AD) (122), AD-related mutations (APP Swedish crossed with Tau VLW) (108) or the *MAPT* mutation P301L alone (123). Tideglusib and lithium considerably reduce phosphorylation and aggregation of tau, decrease neuroinflammation and neuronal death and improve learning and memory abilities (108, 120–123). Both drugs have also been tested in phase I and II clinical trials for AD with overall positive results, demonstrating safety, tolerability and improved cognition in comparison to placebo-treated individuals (124) [reviewed in (125)]. The therapeutic potential of lithium has further been evaluated in the R6/2 mouse model and in HD patients, and studies have reported encouraging results (126–128) [reviewed in (129)]. Lithium was found to regulate HD-associated pathological processes such as glutamate excitotoxicity, altered levels of neurotrophic and growth factors and transcriptional dysregulation (130–133) [reviewed in (129)]. For example, lithium increased levels of brain-derived neurotrophic factor (BDNF) and nerve growth factor (NGF) in several brain regions of an animal model of mania generated with ouabain or wild-type animals, as well as in wild-type cultured neurons (133–136). Furthermore, exposure to lithium mediated significant transcriptional changes connected to signal transduction (e.g., mTOR and Wnt-related signaling) in the corpus callosum of wild-type rats (137), as well as transcription factors and genes related to metabolism in the substantia nigra pars compacta of parkinsonian mice (induced by the neurotoxin MPTP) (138). Clinical studies showed that motor abnormalities were not improved in most HD patients treated with lithium, but some reports suggest an amelioration of cognitive disturbances and mood disorder (139, 140). The positive outcomes on non-motor impairments suggest a potential benefit of lithium treatment in HD individuals, especially for patients in early stages of disease, when cognitive and psychiatric symptoms are predominant. However, it is unclear whether the administration of lithium diminishes GSK-3β activity and p-tau levels, as conflicting findings on GSK-3β levels in post-mortem tissues of HD patients have been reported. Indeed, GSK-3β levels and activity have been shown to be intrinsically decreased in the striatum and cortex of HD individuals (83, 141), while increased GSK-3β levels were measured in the hippocampus (55). These discrepancies may reflect a dynamic molecular response at different disease stages, or suggest the differential alteration of GSK-3β levels in specific brain regions. Evaluation of p-tau in the CSF or imaging with p-tau PET tracers could help identify whether the beneficial effects of lithium are directly related to a reduction of p-tau levels.

#### Phosphatase Activators

Since tau phosphorylation is a reversible process, triggering tau dephosphorylation at serine and threonine residues could reduce hyperphosphorylation. Phosphatases [described extensively in (57)] catalyze the interconversion reactions of tau, from phosphorylated to dephosphorylated states, thereby regulating the degree of tau phosphorylation. Several studies have demonstrated that the activity of protein phosphatase 2A (PP2A) and serine/threonine-protein phosphatase (PP2B) is decreased in AD mouse models and patients (58, 142) [reviewed in (59)], as well as in both Tg and knock-in HD mouse models (56, 64, 143) (Table 1). The Tg mouse line used in the aforementioned studies was the R6/1, which expresses the exon 1 of the human *HTT* gene containing approximately 114 CAG repeats (115) and the R6/2 model, described above. The knock-in mouse lines discussed above were KI140 and zQ175, which respectively express human *HTT* exon 1 with approximately 140 and 188 CAG repeats inserted in the mouse *Htt* (116, 144). Furthermore, PP2A is an important phosphatase known to regulate the activity of the GSK-3β and other kinases implicated in tau pathological modifications (145), and dysregulation of PP2A activity leads to cellular dysfunction including cytoskeletal alterations, impairment of synaptic function and tau mislocalization (146–149). Activation of the phosphatase PP2A has been shown to reduce abnormal phosphorylation of tau in the brain and ameliorate AD pathology (150, 151), suggesting that PP2A phosphatase activators could likely restore physiological levels of tau phosphorylation in patients.

Memantine is a small molecule that has been found to reverse tau hyperphosphorylation. It enhances PP2A activity, improves neuronal viability (152) and reduces glutamate excitotoxicity in GABAergic neurons by acting as a N-methyl-D-aspartate (NMDA) receptor antagonist (153). Furthermore, Memantine mitigates lipopolysaccharide-induced neurodegeneration in mixed rat primary cultures by attenuating the microglial inflammatory response (154). It therefore appears that Memantine is a multifunctional molecule that acts on several cell types and on tau-independent molecular targets, although additional studies are needed to determine if tau directly promotes the beneficial effects of Memantine. Clinical studies indicated that Memantine improves attention, agitation, delusion, global well-being, daily functions and independence in patients with mild cognitive decline (106, 107). There are 87 Memantine clinical trials listed on clinicaltrials.gov, all aiming to treat dementia associated with neurodegenerative diseases, including HD. Thus far, a small pilot (155) and one case study (156) have reported that Memantine improves motor, but not cognitive deficits, in HD patients. However, it may be premature to conclude from these small-scale studies, especially given the fact that both trials were based on the selection of patients in advanced stages of disease that may have further tainted the true potential of Memantine. Hence, Memantine is currently under investigation in the clinical trial MITIGATE-HD, which recruited a large number of pre-manifest HD, early HD and control individuals providing a better opportunity to investigate this compound as a treatment for cognitive impairments. However, the secondary outcome measures of the MITIGATE-HD study do not include CSF t-tau or p-tau nor tau PET imaging evaluation and it will therefore not be possible to determine whether the improvement in cognition, if any, is due to the ability of Memantine to reduce tau hyperphosphorylation.

#### Views on Targeting Pathological Forms of P-Tau

Although targeting the mechanisms of phosphorylation/dephosphorylation of tau is a seemingly logical strategy, several limitations should be considered. Based on the outcomes of AD clinical trials, the major drawback is the dysregulation of essential kinases and phosphatases responsible for the post-translational modifications of unrelated tau substrates. For instance, GSK-3β phosphorylates more than 100 substrates, while PP2A has more than 300 known targets. Furthermore, both enzymes are themselves regulated by a number of signaling pathways (157, 158). Pharmacological alterations of these complex signaling pathways may lead to activation of compensatory mechanisms, which would in turn generate unpredictable outcomes (158). For example, pharmacological activation of the PP2B phosphatase could promote dephosphorylation of mHTT at serine 421, leading to the disruption of axonal transport and cellular distribution of important neurotrophic factors (159). Small molecules aimed at specifically targeting the multi-substrate enzymes involved in tau phosphorylation are accompanied by decisive limitations, which constrain their use in the treatment of HD and other tauopathies. However, the promising clinical results obtained with lithium and Memantine do warrant further investigation in the context of HD.

### Inhibiting the Formation of Tau Aggregates

When hyperphosphorylated tau detaches from the microtubules, it relocates to the somatodendritic compartment and self-assembles into increasingly complex aggregate species. This process begins with soluble oligomers which grow into pre-fibrils and ultimately form insoluble NFTs (66) [reviewed in (160)]. These NFTs accumulate with disease progression in AD (34, 161, 162) as well as in HD (44–46, 99) (Table 1), and therapeutic strategies to prevent tau aggregation have therefore become a major focus of research (Figure 1). However, the perspective that accumulation of NFTs causes neurodegeneration has been more recently challenged (163, 164) [reviewed in (165)]. Indeed, mislocalized, soluble misfolded, soluble hyperphosphorylated forms of tau and tau oligomers are emerging as candidate neurotoxic entities (166–168). For example, the mislocalization of tau to dendritic spines has been established in AD and seemingly induces synaptic impairments in rTg4510 and P301S tau mouse models (167, 169–171) as well as the loss of dendritic spines in AD patients (172). Spinal alterations have been observed in R6/2 mice (173), but the relationship between tau and spine instability in HD remains to be elucidated. Additionally, as observed in AD and HD, pre-tangles and NFTs have been identified in several brain regions, predominantly in the putamen, cortex and hippocampus (22, 27, 43, 44, 46, 98–100). It is therefore reasonable to anticipate that inhibiting tau aggregation at early and late stages of fibrillization may improve HD-related neurotoxicity and, on a larger scale, cognitive deficits.

The design of inhibitors of tau aggregation has already generated a significant number of small molecules for drug screening. Among 3,000 anti-tau aggregation molecules studied, fibrillization inhibitors have been identified as the most effective in preventing the formation of NFTs in Tg animals expressing mutated human tau [reviewed in (174)]. For example, Methylene Blue and its derivatives have been shown to stabilize tau in a conformation that prevents its fibrillization (175), but other neurotoxic forms of tau, such as oligomers, do not appear to be affected (164, 176). Nonetheless, treatment of JNPL3 Tg mice expressing the P301L tau mutation results in the amelioration of cognitive deficits (177) and Methylene Blue is the only tau-targeting treatment that has reached phase III in AD. Importantly, this drug can inhibit the aggregation of other self-assembling proteins including mHTT (178), decrease the formation of mHTT inclusion bodies in primary mouse neurons and R6/2 mice, and improve motor deficits in these animals (178).

#### Views on Inhibiting the Formation of Tau Aggregates

The current state of the field offers histological evidence of tau aggregation in post-mortem HD brain tissue, but available experimental animal models do not recapitulate pathological tau inclusions. Establishing alternative models that reproduce key features and molecular dysfunctions of HD is a prerequisite to evaluate the contributions of tau aggregates to pathology as well as the potential of anti-tau aggregation therapies. Additionally, it is becoming increasingly clear that pathological forms do not solely consist of NFTs, but include aggregate intermediates and soluble post-translationally modified tau. Considering the similarities among tauopathies, an efficient therapeutic approach for HD should therefore target multiple tau species, including oligomers and hyperphosphorylated forms of the protein (27).

### Tau Immunotherapies

Tau-based immunotherapies refer to the neutralization and clearance of the tau protein by host-generated antibodies (active immunization) or by the administration of tau-specific antibodies (passive immunization). Immunotherapies can be designed to target a variety of tau species, including t-tau, hyperphosphorylated tau, extracellular tau, oligomeric tau or tau fragments (Figure 1) (165, 179–181). This approach provides greater precision and flexibility to therapeutic designs, but a successful clinical outcome relies on the identification of the exact pathological forms of tau responsible for a specific phenotype. Multiple forms of tau are potential candidates for immunotherapies in HD, including tau aggregates (27), p-tau (27, 32), tau oligomers (27) and caspase-2 cleaved Δtau314 (84). Each form appears to be associated with distinct disease phenotypes/stages, and tau hyperphosphorylation at S396, S404, T205, and S199 epitopes has been observed in stage 4 HD patients (32). The presence of tau oligomers (T22 and TOMA positive staining) has also been detected in the putamen of stage 4 HD patients (27). Caspase-2 cleaved Δtau314 protein is a form of tau associated with dementia in Lewy body disease (182) and is found in greater concentrations in the caudate nucleus and prefrontal cortex of HD subjects when compared to healthy controls (84). The following paragraphs will survey the available literature to propose additional therapeutic frameworks for the next generation of HD drugs.

#### Active Immunization

Active immunization by exposure of the host immune system to pathological forms of tau has also been considered to induce a long-lasting anti-tau immunity. Several peptides have been designed using tau pathological forms such as p-tau (181, 183), oligomers (180, 184) and truncated tau (185). Immunization of animals recapitulating AD features can result in pathophysiological and behavioral improvements (181), with the host-generated antibodies depict specific recognition of p-tau (181). Based on these findings, two anti-tau vaccines have been tested in AD clinical trials; one targeting S396 and S404 phosphorylated tau (ACI-35) (186) and the other targeting the pathological N-truncated form of tau (AADvac-1 or Axon peptide 108 conjugated to keyhole limpet hemocyanin) (187). Importantly, the ACI-35 vaccine was designed against pathological forms of tau also found in HD patients and further investigations could establish its potential as a candidate immunotherapy to ameliorate cognitive deficits in this patient population (27, 99).

#### Passive Immunization

Passive immunization is achieved by administering pre-formed antibodies to recognize a specific antigen and constitutes an alternative immunization approach that does not solely rely on the immune system of the host. Antibodies can further be engineered to express desirable properties and bind tau within intracellular and/or extracellular compartments. Several antibodies have been designed to target various forms of tau and the promising antibodies that have reached clinical trials are discussed below.

##### Passive immunization to target intracellular tau

Following peripheral administration, anti-tau antibodies are able to cross the blood brain barrier to reach neuronal and non-neuronal elements. The antibodies are internalized by neurons via receptor-mediated endocytosis (188), bind tau and the emerging tau-antibody complex is subsequently degraded through the proteasomal system (189). A significant number of tau passive immunotherapies have been designed to target p-tau, and the anti-tau pS202 monoclonal antibody (named CP13) has thus far demonstrated a superior efficacy compared to other anti-p-tau antibodies (190). Indeed, CP13 reduces soluble and insoluble total and p-tau levels in the cortex and hindbrain of a Tg mouse model expressing the human P301L mutation and which is characterized by a severe tauopathy (190). In HD, increased pS202 levels correlate with alterations in tau phosphorylation in both R6/2 and zQ175 mouse models (56, 64), as well as in patients (27, 42, 43). In addition to p-tau, antibodies have been designed to target other pathological forms of the protein. The humanized antibody LY3303560 (also referred to as Zagotenemab) is derived from a monoclonal antibody used in histology (MC1) to identify pathological conformations of tau (191) in both AD (191) and HD human brain tissue (33). It has also demonstrated greater affinity toward soluble tau aggregates compared to tau monomers *in vitro* (179). This immunotherapy is safe and well-tolerated in humans, as established by the successful completion of a phase I and an ongoing phase II clinical trial evaluating its efficacy in AD (192). CP13 and LY3303560 are therefore attractive immunotherapy candidates that could be tested for efficacy in preclinical models of HD. Animal models such as zQ175 or KI140 mice recapitulate a slow disease progression and the gradual acquisition of molecular and behavioral HD phenotypes, and could therefore provide a suitable experimental system to evaluate the benefits of immunotherapies on HD-related cognitive as well as motor deficits (116, 144).

##### Passive immunization to target extracellular tau

In addition to neurotoxic effects resulting from post-translational modifications, tau has been suggested to adopt prion-like properties that result in its transcellular propagation through the extracellular milieu, to ultimately seed pathology in healthy recipient cells (193). Evidence from cellular (194, 195) and animal models (39–41), as well as post-mortem tissue (36, 161, 162), support this hypothesis (Table 1). For example, a single injection of AD brain homogenate in the cortex or hippocampus of a naïve mouse induces endogenous tau misfolding and aggregation with detrimental consequences on physiological functions and behavior (41). Extracellular tau is particularly efficient in corrupting functional endogenous tau and initiating pathological aggregates that lead to the formation of NFTs (196). A number of mechanisms of tau spreading have been identified and include cytoplasmic exchange by tunneling nanotubes (197), as well as transsynaptic (198) or transcellular propagation via exocytosis and endocytosis (194, 195, 199). Several antibodies targeting extracellular tau have been designed to prevent transcellular propagation and seeding of tau-related pathology. Antibodies are now available to recognize a diversity of extracellular tau isoforms and fragments. The antibody C2N-8E12 (also referred to as ABBV-8E12) recognizes the isoform tau-F (441 aa) (200), while the antibody RO7105705 (also referred to as Semorinemab) binds the N-terminus of all six tau isoforms (201). Gosuranemab (also referred to as BMS-986168) was engineered using a tau fragment released into the conditioned media prepared from AD patient-derived cortical neurons (202, 203). Despite differences in the targeted epitopes, all of these antibodies have been shown to ameliorate tau pathology and behavioral deficits in mouse models that express *MAPT* mutations (JNPL3, P301L and P301S) (203–206). These observations suggest that reducing extracellular levels of tau is a promising strategy to improve AD pathology. Favorable to their use is the fact that antibodies tested in phase I clinical trial have met safety and tolerability criteria (207) [reviewed in (208)]. Similar routes of tau propagation have been suggested to occur in HD patients who received fetal grafts to replace cell loss generated by the disease process (42). However, these observations originate from post-mortem analyses on a few rare cases and more definitive evidence is needed to conclude that tau can indeed propagate in the HD brain. Furthermore, HD patients have lower concentrations of t-tau in the CSF in comparison to AD affected individuals, (96, 97, 209–212), and whether CSF t-tau levels truly reflect extracellular tau load in the central nervous system (CNS) of HD patients remains to be elucidated.

#### Views on Tau Immunotherapies

Research in the field of immunotherapy has made significant progress in the development of anti-tau treatments for AD and is gaining momentum in HD [reviewed in (101, 213)]. Antibody-based therapies have the distinct advantage of being highly specific to the selected epitopes and can target intracellular or extracellular tau both in the periphery and in the CNS. Antibodies have a low molecular weight and are therefore suitable for nanocarrier-based delivery approaches to reduce their degradation, mitigate the host immune response and control the rate of antibody release (214). Furthermore, administration of tau-targeting antibodies by injection is a straightforward and safe procedure (207). Based on the concept that mHTT can propagate between cells and template pathology in a prion-like fashion, active and passive immunization strategies targeting extracellular mHTT are attracting interest (215–222) [extensively reviewed in (213)], and could be tested in parallel to a tau, or mHTT/tau combined immunization. Furthermore, halting accumulation and propagation of pathogenic proteins in HD patients at early stages of the disease, before they are afflicted by neurodegeneration and cognitive dysfunction, may provide the most effective protection.

However, a major limitation of current studies is that the majority of tau-related dysregulations have not been reported in premanifest or early-stage diseased patients. As a result, the association of pathological forms of tau with late stage disease could cast doubts on the validity of targeting tau in HD (32). A broad investigation on a larger population of HD patients, matched for age, sex and CAG repeat length, would provide more accurate information on the progression of tau pathology. In particular, future studies could take advantage of the technological advances achieved with PET scans and specific anti-tau ligands (223), to enable the analysis of pathological tau in the brain of living patients with a degree of specificity that is not possible with post-mortem analyses. By doing so, tau abnormalities could be directly compared to cognitive performance and importantly, changes over time. For instance, the PET ligand [^18^F]MK-6240, which has already been validated in AD, is a new generation tau ligand with high sensitivity for NFTs and negligible off-target binding (224), which makes it ideal for defining the extent of tau pathology in HD brains and investigating its relationship with cognitive profile.

## Mitigation of Tau-Induced Cellular Alterations by Stabilizing Microtubules

Structural and functional damage to the neuronal cytoskeleton constitutes a key event in the pathogenesis of tauopathies (225–227). Therefore, microtubule-stabilizing small molecules have been developed and studied as a therapeutic approach to treat tauopathies (Figure 1). Alterations of microtubule-dependent axonal transport is a characteristic of HD pathology (78, 79) and could result from the independent, but coordinated effects of mHTT and tau (Table 1). mHTT has been shown to interact with and destabilize microtubules, resulting in alterations of axonal transport and loss of neuronal viability (78, 79, 228). Tau has further been found to recruit mHTT to the microtubule network (56), which could precipitate the destabilization of microtubules. For example, microtubule stabilization using the small molecule Taxol, a semisynthetic taxane derivative, inhibits the entry of mHTT into the nucleus and increases neuronal survival in HD primary striatal and cortical neurons (78). These observations suggest that tau and mHTT can both induce microtubule destabilization, which support the restoration of microtubule functions as a therapeutic approach for HD.

The cytoskeleton stabilizing agents Epothilone and Taxane derivatives have been found to interact with tubulin, restore rapid axonal transport, and alleviate cognitive and motor impairments in mouse models of tauopathies (T44 tau Tg mice; PS19 tau Tg mice) (71, 229). However, the Epothilone D phase I clinical trial was discontinued due to toxicity and severe side-effects were reported for taxoid TPI 287 (abeotaxane) (230). On the other hand, the microtubule-stabilizing peptide Davunetide (NAP) (231, 232) was well-tolerated in preclinical toxicology and clinical safety studies (233). NAP demonstrated highly potent neuroprotective properties by reducing the activity of the microtubule-severing protein katanin (234), inhibiting programmed cell death and restoring mitochondrial function in the PD-related A53T α-synuclein SH-SY5Y cell culture model (235). In a Tg schizophrenia mouse model (activity-dependent neuroprotective protein (ADNP)^+/−^ Tg mice), intranasal administration of NAP decreased brain levels of p-tau and improved cognition (236). The treatment of patients suffering from mild cognitive impairments with NAP showed improved attention and working memory (233). However, no improvement of cognitive deficits was observed in Progressive Supranuclear Palsy (PSP) patients treated with NAP, which could be partly explained by an ineffective dosage (237).

### Views on Stabilizing Microtubules

The regulation and restoration of microtubule function is an attractive strategy substantiated by promising preclinical results. Epothilone and Taxane derivatives were approved by the FDA in the early 1990s [history reviewed in (238)] as chemotherapies to treat aggressive cancers and despite significant side-effects, they are among the most relied upon therapies to treat solid tumors (239, 240). In the case of neurodegenerative diseases, the toxicity of microtubule-stabilizing agents is a major drawback that may limit their clinical use, altogether. However, new microtubules stabilizing agents such as Davunetide and others [reviewed in (241)] exhibit promising disease-mitigating outcomes in animal models, with more manageable side-effects in clinical trials, and could therefore be the new frontier in the search for microtubules stabilizing strategies to ameliorate tau pathology in neurodegenerative diseases, including HD.

## Modulation of *MAPT* Gene Expression

Mutations in the *MAPT* gene have been associated with neurodegenerative diseases such as Frontotemporal dementia (20, 242–245), PSP (245) and AD (17–21); diseases characterized by abnormal accumulation of pathological forms of tau and that further correlate with cognitive deficits. Impaired alternative splicing of exon 10 of the *MAPT* gene leads to an imbalance in the amount of 3R and 4R tau isoforms within cells, and a change in 3R to 4R ratio has been associated with specific tauopathies. For instance, NFTs are found to be immunopositive for both 4R and 3R in AD (28), while patients with Pick's disease predominantly express the 3R tau isoform (246). As a result, temporarily altering *MAPT* expression or splicing using antisense oligonucleotides (ASO) has been considered (Figure 1) (247). To our knowledge, no *MAPT* mutations have been associated with HD, but patients express lower 3R and increased 4R tau mRNA and protein levels (32) and the 4R isoform is enriched in tau nuclear rods found in the striatum (31). Therefore, selectively reducing the levels of 4R tau without altering the overall expression of tau protein in HD could restore protein homeostasis (31, 32).

Tau is involved in essential cellular functions (248) and as a result, tau silencing could raise safety concerns that would limit its translation to the clinic. Studies in tau knock-out animal models have demonstrated that loss of *Mapt* causes cognitive and motor deficits in an age- and strain-dependent manner (249). A marked reduction in the number of dopaminergic neurons was observed in the substantia nigra pars compacta of *Mapt* ^−/−^ mice, which correlated with PD-like motor deficits in 12-month old animals (249, 250). However, the partial or total downregulation of the *Mapt* gene in the R6/1 Tg (115 CAG repeats) HD mouse model improves motor behavior and does not induce significant side-effects (31). Furthermore, the reduction of tau protein levels using the ASO BIIB080 prevents NFTs deposition and clears pre-existing tau aggregates, mitigates neuronal loss, ameliorates behavior (nesting) and extends the survival of PS19 Tg mice expressing the disease-associated P301S tau mutation (251). In non-human primates, ASO BIIB080 reduces tau mRNA and protein levels in the brain, spinal cord and CSF (251). These promising results enabled the advancement of ASO BIIB080 to phase I clinical trial that is currently ongoing (252).

### Views on Modulating *MAPT* Gene Expression

Modulation of gene expression is a novel therapeutic avenue to treat tauopathies and mRNA-targeting strategies using ASO have recently been proposed as a potential treatment for HD (253). The preliminary results of a clinical trial on IONIS-HTTRx, an ASO drug that targets *HTT*, reported a sharp decrease in mHTT CSF levels with no serious adverse events (254, 255). To our knowledge, silencing more than one gene via ASOs has never been tested, but lowering both HTT and tau expression levels in HD patients has recently been proposed (256). However, gene silencing and modification of gene expression are novel technologies with only a few treatments approved for clinical use. For instance Fomivirsen, an antisense antiviral drug for the treatment of retinitis cytomegalovirus, was the first antisense drug approved by the FDA in 1998 (257), and the second ASO therapy to reach the market (Mipomersen, to treat familial hypercholesterolemia) was FDA approved only 15 years later. As of today, little is known about the possible long-term consequences of modulating protein levels with ASOs, and it is essential to extensively investigate whether modulation of both *MAPT* and *HTT* expression could ameliorate disease outcome in HD patients without inducing long-lasting side-effects.

## Additional Tau-Targeting Approaches

In addition to phosphorylation, tau can undergo a number of post-translational modifications such as glycosylation (9), acetylation (7), truncation (258), glycation (259), nitration (260) and ubiquitination (261) [reviewed in (262)]. These modifications have been associated with tauopathies and mainly investigated in AD brains, with observation of increases in acetylated (7), caspase-truncated (263) and N-glycosylated forms of tau (9). Several molecules have been tested in preclinical and clinical studies to target these different disease-associated post-translational modifications. For example, Salsalate is a nonsteroidal anti-inflammatory drug that decreases t-tau levels and acetylated tau at the K174 residue, ameliorates hippocampal atrophy and memory deficits in PS19 Tg mice (8), and has consequently advanced to phase 1 PSP clinical trial (264). It remains undetermined whether tau post-translational modifications, such as acetylation or glycosylation, occur in HD and only a broader understanding of all forms of tau alterations in the disease would support the exploration of these additional tau-targeting approaches.

Another aspect of pathology is the impairment of protein degradation via alterations of the UPS, which has been observed and associated with tauopathies, and proposed to mediate the accumulation of tau within cells (265, 266) [reviewed in (267)]. The search for drugs that increase the degradation of tau by activating the UPS led to the identification of Rolipram (268). Rolipram has been found to activate the UPS by stimulating the protein kinase A (PKA)/cAMP pathway in *ex vivo* cortical brain slices of rTg4510 mice and to reduce both t-tau and insoluble tau levels (266). Rolipram also decreases t-tau and p-tau levels in a Tg mouse model of early-stage tauopathy expressing the proteasome 26S-targeted fragment (rTg4510:Ub-G76V-GFP) and improves reversal learning behavior in experimental animals (266).

Disease-associated post-translational modifications of tau, as well as dysfunction of the UPS, are compelling alternatives to explore for the treatment of tauopathies. In contrast to the therapeutic strategies discussed in sections *Therapeutic Strategies to Target Pathological Forms of Tau, Mitigation of tau-induced cellular alterations by stabilizing microtubules, Modulation of MAPT Gene Expression* of this review, the relevance of these pathways remains to be demonstrated in models of HD, and the functional and mechanistic contributions of UPS dysfunction to HD pathology are unclear (269). HD-related alterations of the UPS have been hinted at, with early studies showing sequestration of UPS components within mHTT inclusion bodies (87, 270) in Tg HD mouse models (R6/1, R6/2 and R6/5 Tg - 130-155 CAG repeats) (87) as well as in human brain tissue (270). However, further characterization suggested that UPS impairments could be a transient phenotype, as global UPS activity was not significantly impaired in the R6/2 model and in the HD94 mouse model that expresses mHTT fragments under a tetracycline-responsive system (271, 272). An explanation for these seemingly contradictory findings was proposed by Schipper-Krom et al. who suggested that proteasome recruitment into inclusion bodies is a dynamic and reversible process, that does not inhibit the catalytic activity of the sequestered proteasome units (273), thus supporting observations that the UPS activity may not be impaired in HD mouse models. Nonetheless, pharmacological and genetic modulation of the proteasome activity suggest its implication in mHTT aggregation and cell survival, as a reduced activity is associated with increased aggregation (88, 274) and increased activity promotes the survival of HD striatal neurons (275).

## Conclusion

HD patients and their families carry a heavy emotional and financial burden as they face the challenges of a very complex disease, which manifests with a blend of motor, neuropsychiatric and cognitive deficits. Efficient symptomatic treatments, and more importantly disease-modifying therapies, are urgently needed. The diversity of symptoms and difficulty to target the root of the disease, a mutation in the *HTT* gene, suggests that combinatorial therapies that attenuate multiple dysfunctional proteins and pathways may ultimately be the best strategy to tackle the complex portrait of HD (Table 1). Here, we have reviewed compelling evidence suggesting that HD patients develop features of tauopathies and tau-related dysfunctions. These include the (i) altered exon 10 splicing of *MAPT* (31, 32), (ii) correlations between H2 *MAPT* haplotype and severity of cognitive decline (27), (iii) dysfunction of tau-targeting kinases and phosphatases (55, 56, 64, 83), (iv) increased soluble hyperphosphorylated tau (32), (v) altered levels of t-tau in the brain and CSF (32, 96, 97, 209), and (vi) presence of brain NFTs (27, 98, 99). The accumulating evidence of tau pathology encompasses broad dysregulations at the genetic and molecular levels, in CNS tissue and associated biological fluids, and therefore argue in favor of the classification of HD as a secondary tauopathy. In-depth studies are now needed to determine if tau dysfunction directly causes some of the features associated with HD, and in particular cognitive deficits.

Therapeutic approaches targeting tau pathology have been initially designed to treat AD (Figure 1) (101) or primary tauopathies such as PSP (276), based on a large body of evidence suggesting a strong connection between alterations of tau function and cognitive decline [extensively reviewed in (277)]. HD pathology is also associated with both pathological forms of tau and cognitive impairments, thus tau-targeting therapies may offer a completely new angle to treat HD-associated cognitive dysfunction. A diversity of tau-targeting methodologies has been developed, from small molecules to immunotherapies and modulation of gene expression using ASO. These therapies are at various stages of drug development, providing exciting and hopeful prospects toward the betterment of the HD condition.

## Author Contributions

MM and SS contributed equally to reviewing the literature and writing the first draft of the manuscript. ARJ, MA, and FC contributed to discussions regarding the manuscript, revised, and finalized the review. All authors contributed to the article and approved the submitted version.

## Conflict of Interest

The authors declare that the research was conducted in the absence of any commercial or financial relationships that could be construed as a potential conflict of interest.
